# Supplementary material for: Three-Dimensional Topological States of Phonons with Tunable Pseudospin Physics
Source: Research (Wash D C). 2019 Jul 31;2019:5173580. doi: 10.34133/2019/5173580 (PMC6750063; doi:10.34133/2019/5173580)
Supplement: Supplementary Materials — (I) Derivation of effective Hamiltonians, (II) topological phase diagram, (III) interatomic coupling parameters, (IV) hybrid Wannier centers, (V) pseudospin textures, (VI) phonon transport in tunneling junctions, (VII) influence of out-of-plane vibrations, and (VIII) phononic topological semimetal. [file 5173580.f1.pdf]

Supplemental Material on "Three-dimensional topological states of phonons with tunable pseudospin physics"

Yizhou Liu[1,2,3], Yong Xu[1,2,3,*] and Wenhui Duan[1,2,4]

[1]*State Key Laboratory of Low Dimensional Quantum Physics, Department of Physics, Tsinghua University, Beijing 100084, People's Republic of China*

[2]*Collaborative Innovation Center of Quantum Matter, Beijing 100084, People's Republic of China*

[3]*RIKEN Center for Emergent Matter Science (CEMS), Wako, Saitama 351-0198, Japan*

[4]*Institute for Advanced Study, Tsinghua University, Beijing 100084, People's Republic of China*

Contents

I. Effective Hamiltonians 1

II. Topological Phase diagram 3

III. Interatomic coupling parameters 3

IV. Hybrid Wannier centers 4

V. Pseudospin textures 5

VI. Phonon transport in tunneling junctions 7

VII. Influence of out-of-plane vibrations 8

VIII. Phononic topological semimetal 9

I. EFFECTIVE HAMILTONIANS

We derive the type-I effective Hamiltonian first. We choose the basis as $\{|\mathrm{I}_\uparrow\rangle, |\mathrm{O}_\uparrow\rangle, |\mathrm{I}_\downarrow\rangle, |\mathrm{O}_\downarrow\rangle\}$:

$$|\mathrm{I}_\uparrow\rangle = (\varepsilon_+ \sin\theta_\mathbf{k}, \varepsilon_+ \cos\theta_\mathbf{k})^T,\ |\mathrm{O}_\uparrow\rangle = (\varepsilon_- \cos\theta_\mathbf{k}, -\varepsilon_- \sin\theta_\mathbf{k})^T,$$
$$|\mathrm{I}_\downarrow\rangle = i(\varepsilon_- \sin\theta_\mathbf{k}, \varepsilon_- \cos\theta_\mathbf{k})^T, |\mathrm{O}_\downarrow\rangle = i(-\varepsilon_+ \cos\theta_\mathbf{k}, \varepsilon_+ \sin\theta_\mathbf{k})^T,$$

where $\varepsilon_\pm = (1, \pm i)/\sqrt{2}$, and the vibrational magnitude of each atomic site is determined by $\theta_\mathbf{k} \in [0, \pi/2]$. The diagonal matrix element $\langle \mathrm{I}_\uparrow|H|\mathrm{I}_\uparrow\rangle$ transforms under C_3 as:

$$C_3 : \langle \mathrm{I}_\uparrow|H_\mathbf{k}|\mathrm{I}_\uparrow\rangle = \langle \mathrm{I}_\uparrow|C_3^{-1}C_3H_\mathbf{k}C_3^{-1}C_3|\mathrm{I}_\uparrow\rangle = \langle \mathrm{I}_\uparrow|e^{-i\frac{2\pi}{3}}H_{C_3\mathbf{k}}e^{i\frac{2\pi}{3}}|\mathrm{I}_\uparrow\rangle = \langle \mathrm{I}_\uparrow|H_{C_3\mathbf{k}}|\mathrm{I}_\uparrow\rangle, \tag{S1}$$

which is invariant under C_3. The C_3 invariant terms up to $O(k^2)$ are of the form $M^I_\mathbf{k} = \omega^I - A^I k_z - B^I_1(k_x^2 + k_y^2) - B^I_2 k_z^2$, where ω^I, A^I, B^I_1 and B^I_2 are real constants. In addition, the system has mirror symmetries $\sigma_{v(d)}$ (Fig. S1) and time-reversal symmetry. The linear term $A^I k_z$ breaks the combined $\sigma_d T$ symmetry so it should not exist. Furthermore, by taking into account of the mirror symmetry σ_v:

$$\sigma_v : \langle \mathrm{I}_\uparrow|H_\mathbf{k}|\mathrm{I}_\uparrow\rangle = \langle \mathrm{I}_\uparrow|\sigma_v^{-1}\sigma_v H_\mathbf{k}\sigma_v^{-1}\sigma_v|\mathrm{I}_\uparrow\rangle = \langle \mathrm{I}_\downarrow|H_{\sigma_v\mathbf{k}}|\mathrm{I}_\downarrow\rangle. \tag{S2}$$

*Electronic address: yongxu@mail.tsinghua.edu.cn

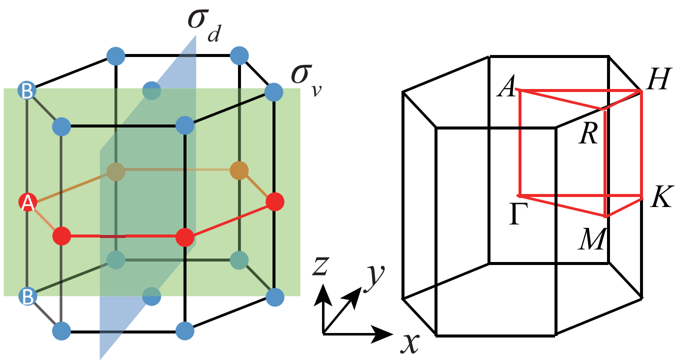

FIG. S1: Two different mirror symmetries of C_{6v} group: σ_v and σ_d. For type-I model at K or H, σ_v is a symmetry element, but σ_d is not. However, the combined $\sigma_d T$ is a symmetry element, where T refers to time-reversal symmetry.

Similarly, the other two diagonal matrix elements are: $\langle \mathrm{O}_\uparrow|H_\mathbf{k}|\mathrm{O}_\uparrow\rangle = \langle \mathrm{O}_\downarrow|H_\mathbf{k}|\mathrm{O}_\downarrow\rangle = \omega^O - A^O k_z - B_1^O(k_x^2+k_y^2) - B_2^O k_z^2$. Therefore, the diagonal matrix elements are written as:

$$\begin{aligned}
\mathrm{diag}(H) &= \begin{pmatrix} \omega^I - B_1^I(k_x^2+k_y^2) - B_2^I k_z^2 & 0 & 0 & 0 \\ 0 & \omega^O - B_1^O(k_x^2+k_y^2) - B_2^O k_z^2 & 0 & 0 \\ 0 & 0 & \omega^I - B_1^I(k_x^2+k_y^2) - B_2^I k_z^2 & 0 \\ 0 & 0 & 0 & \omega^O - B_1^O(k_x^2+k_y^2) - B_2^O k_z^2 \end{pmatrix} \\
&= \omega_\mathbf{k}\mathbb{I}_{4\times4} + \begin{pmatrix} M_\mathbf{k} & 0 & 0 & 0 \\ 0 & -M_\mathbf{k} & 0 & 0 \\ 0 & 0 & M_\mathbf{k} & 0 \\ 0 & 0 & 0 & -M_\mathbf{k} \end{pmatrix}, \\
\omega_\mathbf{k} &= \omega_0 - \tilde{B}_1(k_x^2+k_y^2) - \tilde{B}_2 k_z^2, \\
M_\mathbf{k} &= M - B_1(k_x^2+k_y^2) - B_2 k_z^2,
\end{aligned} \tag{S3}$$

where $\omega_0 = \frac{1}{2}(\omega^I + \omega^O)$, $\tilde{B}_1 = \frac{1}{2}(B_1^I + B_1^O)$, $\tilde{B}_2 = \frac{1}{2}(B_2^I + B_2^O)$, $M = \frac{1}{2}(\omega^I - \omega^O)$, $B_1 = \frac{1}{2}(B_1^I - B_1^O)$, and $B_2 = \frac{1}{2}(B_2^I - B_2^O)$. The $\omega_\mathbf{k}\mathbb{I}_{4\times4}$ term has no influence on the topological properties.

Next we consider the possible form of off-diagonal matrix elements. For the matrix element $\langle \mathrm{I}_\uparrow|H_\mathbf{k}|\mathrm{O}_\uparrow\rangle$, it transforms under C_3 as:

$$C_3 : \langle \mathrm{I}_\uparrow|H_\mathbf{k}|\mathrm{O}_\uparrow\rangle = \langle \mathrm{I}_\uparrow|C_3^{-1}C_3 H_\mathbf{k} C_3^{-1} C_3|\mathrm{O}_\uparrow\rangle = \langle \mathrm{I}_\uparrow|e^{-i\frac{2\pi}{3}} H_{C_3\mathbf{k}} e^{-i\frac{2\pi}{3}}|\mathrm{O}_\uparrow\rangle = e^{-i\frac{4\pi}{3}}\langle \mathrm{I}_\uparrow|H_{C_3\mathbf{k}}|\mathrm{O}_\uparrow\rangle = e^{i\frac{2\pi}{3}}\langle \mathrm{I}_\uparrow|H_{C_3\mathbf{k}}|\mathrm{O}_\uparrow\rangle, \tag{S4}$$

which acquires a phase factor of $e^{i\frac{4\pi}{3}}$ or $e^{-i\frac{2\pi}{3}}$. Thus, $\langle \mathrm{I}_\uparrow|H_\mathbf{k}|\mathrm{O}_\uparrow\rangle = \Delta_1(k_x+ik_y) + \Delta_1'(k_x-ik_y)^2 + \cdots = \Delta_1 k_+ + O(k^2)$. On the other hand, the operation of the combined symmetry $\sigma_d T$ acts on the states as $\sigma_d T|\mathrm{I}_\uparrow\rangle = -|\mathrm{I}_\uparrow\rangle$, $\sigma_d T|\mathrm{O}_\uparrow\rangle = -|\mathrm{O}_\uparrow\rangle$. Thus,

$$\sigma_d T : \langle \mathrm{I}_\uparrow|H_\mathbf{k}|\mathrm{O}_\uparrow\rangle = \langle \mathrm{I}_\uparrow|(\sigma_d T)^{-1}\sigma_d T H_\mathbf{k}(\sigma_d T)^{-1}\sigma_d T|\mathrm{O}_\uparrow\rangle = \langle \mathrm{I}_\uparrow|H_{\sigma_d T\mathbf{k}}|\mathrm{O}_\uparrow\rangle^*. \tag{S5}$$

This requires the parameters Δ_1 and Δ_1' to be real. Since under the mirror symmetry σ_v: $|\mathrm{I}_\uparrow\rangle \to -i|\mathrm{I}_\downarrow\rangle$ and $|\mathrm{O}_\uparrow\rangle \to i|\mathrm{O}_\downarrow\rangle$, we have $\langle \mathrm{I}_\downarrow|H_\mathbf{k}|\mathrm{O}_\downarrow\rangle = -\langle \mathrm{I}_\uparrow|H_{\sigma_v\mathbf{k}}|\mathrm{O}_\uparrow\rangle$. Other matrix elements can be derived in a similar way, giving $\langle \mathrm{I}_\uparrow|H_\mathbf{k}|\mathrm{I}_\downarrow\rangle = -iC_1k_+ - iC_1'k_-^2 + \cdots = -iC_1k_+ + O(k^2)$, $\langle \mathrm{O}_\uparrow|H_\mathbf{k}|\mathrm{O}_\downarrow\rangle = iC_2k_-$, and $\langle \mathrm{I}_\uparrow|H_\mathbf{k}|\mathrm{O}_\downarrow\rangle = \Delta_2 k_z + O(k^2)$, where C_1, C_1', C_2, C_2' and Δ_2 are real constants. Considering the Hermitian nature of Hamiltonian, the full matrix form of type-I effective Hamiltonian can be written as:

$$H_\mathbf{k} = \begin{pmatrix} M_\mathbf{k} & \Delta_1 k_+ & -iC_1k_+ & \Delta_2 k_z \\ \Delta_1 k_- & -M_\mathbf{k} & \Delta_2 k_z & iC_2 k_- \\ iC_1 k_- & \Delta_2 k_z & M_\mathbf{k} & -\Delta_1 k_- \\ \Delta_2 k_z & -iC_2 k_+ & -\Delta_1 k_+ & -M_\mathbf{k} \end{pmatrix}. \tag{S6}$$

For the type-II effective Hamiltonian, the symmetry group is C_{6v}, whose symmetry elements doubles those of C_{3v} due to the existence of C_2 (or C_6) rotation symmetry. Since all the basis states $|\mathrm{I(O)}_{\uparrow(\downarrow)}\rangle$ are odd under C_2 rotation, the matrix elements coupling between them should be even under C_2. Thus, all the matrix elements which are linear in k_x or k_y are forbidden. This

result in the type-II effective Hamiltonian as:

$$H'_{\mathbf{k}} = \begin{pmatrix} M_{\mathbf{k}} & \delta_1 k_-^2 & -ic_1 k_-^2 & \delta_2 k_z \\ \delta_1 k_+^2 & -M_{\mathbf{k}} & \delta_2 k_z & ic_2 k_+^2 \\ ic_1 k_+^2 & \delta_2 k_z & M_{\mathbf{k}} & -\delta_1 k_+^2 \\ \delta_2 k_z & -ic_2 k_-^2 & -\delta_1 k_-^2 & -M_{\mathbf{k}} \end{pmatrix}. \tag{S7}$$

II. TOPOLOGICAL PHASE DIAGRAM

We discuss type-I phononic topological insulators (TIs) first. In the inverted regime without H_I and H_{RD} the band crossing occurs at $\sqrt{k_x^2 + k_y^2} = \sqrt{M/B_1} = k_c$ in $k_z = 0$ plane. When H_I and H_{RD} are taken into account the energy levels at k_c are determined by the equation $\det[E\mathbb{I}_{4\times 4} - H(k_c)]=0$, where

$$H(k_c) = \begin{pmatrix} 0 & \Delta_1 k_c & -iC_1 k_c & 0 \\ \Delta_1 k_c & 0 & 0 & iC_2 k_c \\ iC_1 k_c & 0 & 0 & -\Delta_1 k_c \\ 0 & -iC_2 k_c & -\Delta_1 k_c & 0 \end{pmatrix}. \tag{S8}$$

The solution to the eigenvalue equation is determined by $E = \pm\sqrt{A \pm B} k_c$, where

$$\begin{aligned} A &= \Delta_1^2 + \frac{C_1^2 + C_2^2}{2}, \\ B &= |C_1 + C_2| \sqrt{\Delta_1^2 + \frac{(C_1 - C_2)^2}{4}}. \end{aligned} \tag{S9}$$

The topological phase transition occurs at the point where the middle two energy levels cross, i.e., $A - B = 0$. This condition can be solved as $\Delta_1^2 = C_1 C_2$. When $\Delta_1^2 > C_1 C_2$, the pseudo-SOC term $\Delta_1 k_\pm$ dominates, and the band structure can be adiabatically connected to the case of $C_1 = C_2 = 0$ without closing the band gap. Then the effective Hamiltonian reduces to the 3D Bernevig-Hughes-Zhang (BHZ) model that describes a strong topological insulator phase; when $\Delta_1^2 < C_1 C_2$ the band structure can be adiabatically connected to the case of $\Delta_1 = 0$, which results in a topological semimetal (TSM) phase.

The discussion of type-II phononic TIs is quite similar. The system is a type-II phononic TI (TSM) when $\delta_1^2 > c_1 c_2$ ($\delta_1^2 < c_1 c_2$).

III. INTERATOMIC COUPLING PARAMETERS

For the lattice in Fig. S1, we consider the interatomic interactions as $V = \sum_n V_n^A + V_n^B + V_n^{AB}$, where

$$\begin{aligned} V_n^\alpha &= \sum_{<i,j>} \frac{1}{2} k_{1\alpha}^L (\mathbf{u}_{n\alpha\alpha}^{ij} \cdot \mathbf{e}_{ij})^2 + \frac{1}{2} k_{1\alpha}^T (\mathbf{u}_{n\alpha\alpha}^{ij} \times \mathbf{e}_{ij})^2, \\ V_n^{AB} &= \sum_i \frac{1}{2} k_{1AB}^L (\mathbf{u}_{nAB}^{ii} \cdot \mathbf{e}_{ii}^{AB})^2 + \frac{1}{2} k_{1AB}^T (\mathbf{u}_{nAB}^{ii} \times \mathbf{e}_{ii}^{AB})^2 + \frac{1}{2} k_{1BA}^L (\mathbf{u}_{nBA}^{\prime ii} \cdot \mathbf{e}_{ii}^{BA})^2 + \frac{1}{2} k_{1BA}^T (\mathbf{u}_{nBA}^{\prime ii} \times \mathbf{e}_{ii}^{BA})^2, \\ &+ \sum_{<i,j>} \frac{1}{2} k_{2AB}^L (\mathbf{u}_{nAB}^{ij} \cdot \mathbf{e}_{ij}^{AB})^2 + \frac{1}{2} k_{2AB}^T (\mathbf{u}_{nAB}^{ij} \times \mathbf{e}_{ij}^{AB})^2 + \frac{1}{2} k_{2BA}^L (\mathbf{u}_{nBA}^{\prime ij} \cdot \mathbf{e}_{ij}^{BA})^2 + \frac{1}{2} k_{2BA}^T (\mathbf{u}_{nBA}^{\prime ij} \times \mathbf{e}_{ij}^{BA})^2. \end{aligned} \tag{S10}$$

n labels the unit cell along the z-axis. $\alpha = A, B$ labels the sublattice. i and j label the unit cell along the in-plane directions. "<>" indicates the nearest unit cells along the in-plane directions. $\mathbf{u}_{n\alpha\beta}^{ij} = \mathbf{u}_{n\alpha}^i - \mathbf{u}_{n\beta}^j$ and $\mathbf{u}_{n\alpha\beta}^{\prime ij} = \mathbf{u}_{n+1,\alpha}^i - \mathbf{u}_{n\beta}^j$ are the interatomic displacements. $\mathbf{e}_{n\alpha\beta}^{ij}$ and $\mathbf{e}_{n\alpha\beta}^{\prime ij}$ are the unit vectors along the two interacting sites.

With the interatomic interactions in Eq. (S10), the dynamical matrix can be derived as (using the same atomic masses for the

two sites $m_A = m_B = 1$ for simplicity):

$$
\begin{aligned}
D_{\mathbf{k}} &= \begin{pmatrix} D_A & D_{AB} \\ D_{AB}^{\dagger} & D_B \end{pmatrix}, \\
D_A &= \left[\frac{1}{2}(k_A^L + k_A^T)\mathbb{I}_{2\times 2} + \frac{1}{2}(k_A^L - k_A^T)\sigma_z\right](2 - 2\cos k_x a) + \left[\frac{1}{2}(k_A^L + k_A^T)\mathbb{I}_{2\times 2} - \frac{1}{4}(k_A^L - k_A^T)\sigma_z\right](4 - 4\cos\frac{k_x a}{2}\cos\frac{\sqrt{3}k_y a}{2}) \\
&+ \sqrt{3}(k_A^L - k_A^T)\sigma_x \sin\frac{k_x a}{2}\sin\frac{\sqrt{3}k_y a}{2} + \left[k_{1AB}^T + k_{1BA}^T + 6k_{2AB}^T + 6k_{2BA}^T + 3(\tilde{k}_{2AB}^L - \tilde{k}_{2AB}^T) + 3(\tilde{k}_{2BA}^L - \tilde{k}_{2BA}^T)\right]\mathbb{I}_{2\times 2}, \\
D_B &= \left[\frac{1}{2}(k_B^L + k_B^T)\mathbb{I}_{2\times 2} + \frac{1}{2}(k_B^L - k_B^T)\sigma_z\right](2 - 2\cos k_x a) + \left[\frac{1}{2}(k_B^L + k_B^T)\mathbb{I}_{2\times 2} - \frac{1}{4}(k_B^L - k_B^T)\sigma_z\right](4 - 4\cos\frac{k_x a}{2}\cos\frac{\sqrt{3}k_y a}{2}) \\
&+ \sqrt{3}(k_B^L - k_B^T)\sigma_x \sin\frac{k_x a}{2}\sin\frac{\sqrt{3}k_y a}{2} + \left[k_{1AB}^T + k_{1BA}^T + 6k_{2AB}^T + 6k_{2BA}^T + 3(\tilde{k}_{2AB}^L - \tilde{k}_{2AB}^T) + 3(\tilde{k}_{2BA}^L - \tilde{k}_{2BA}^T)\right]\mathbb{I}_{2\times 2}, \\
D_{AB} &= -\left[k_{1AB}^T e^{ik_z c} + 2k_{2AB}^T e^{ik_z c}(\cos k_x a + 2\cos\frac{k_x a}{2}\cos\frac{\sqrt{3}k_y a}{2})\right]\mathbb{I}_{2\times 2} - (\tilde{k}_{2AB}^L - \tilde{k}_{2AB}^T)e^{ik_z c}\left[(\cos k_x a + 2\cos\frac{k_x a}{2}\cos\frac{\sqrt{3}k_y a}{2})\mathbb{I}_{2\times 2}\right. \\
&\left. +(\cos k_x a - \cos\frac{k_x a}{2}\cos\frac{\sqrt{3}k_y a}{2})\sigma_z - \sqrt{3}\sin\frac{k_x a}{2}\sin\frac{\sqrt{3}k_y a}{2}\sigma_x\right] - \left[k_{1BA}^T + 2k_{2BA}^T(\cos k_x a + 2\cos\frac{k_x a}{2}\cos\frac{\sqrt{3}k_y a}{2})\right]\mathbb{I}_{2\times 2} \\
&- (\tilde{k}_{2AB}^L - \tilde{k}_{2AB}^T)\left[(\cos k_x a + 2\cos\frac{k_x a}{2}\cos\frac{\sqrt{3}k_y a}{2})\mathbb{I}_{2\times 2} + (\cos k_x a - \cos\frac{k_x a}{2}\cos\frac{\sqrt{3}k_y a}{2})\sigma_z - \sqrt{3}\sin k_x a \sin\frac{\sqrt{3}}{2}k_y a\sigma_x\right].
\end{aligned}
\tag{S11}
$$

σ_x and σ_z are Pauli matrices, $\mathbb{I}_{2\times 2}$ is the identity matrix, and $\tilde{k}^b_{1(2)AB} = k_{1(2)AB}/[1 + (c_1/a)^2]$, $\tilde{k}^b_{1(2)BA} = k_{1(2)BA}/[1 + (c_2/a)^2]$ with $b = L, T$ refers to the longitudinal or transverse force constants. According to Eq. (S11) the block D_A, D_B, and D_{AB} at the high symmetry points Γ, K, M, A, H, and R are given in Tab. S1.

At the high symmetry points K, H, Γ, and A, the dynamical matrix can be expressed as:

$$
D = \begin{pmatrix} d_A & d_{AB} \\ d_{AB} & d_B \end{pmatrix} \otimes \mathbb{I}_2, \tag{S12}
$$

where d_A, d_B, and d_{AB} are real numbers. The solution to the secular equation $D|\mathbf{u}\rangle = \omega^2|\mathbf{u}\rangle$ is given by

$$
\omega_{\pm}^2 = \frac{1}{2}(d_A + d_B) \pm \sqrt{\frac{1}{4}(d_A - d_B)^2 + d_{AB}^2}, \qquad |\mathbf{u}_{\pm}^s\rangle = C\begin{pmatrix} d_{AB}\varepsilon_s \\ \left[\pm\sqrt{\frac{1}{4}(d_A - d_B)^2 + d_{AB}^2} - \frac{1}{2}(d_A - d_B)\right]\varepsilon_s \end{pmatrix} = C\begin{pmatrix} u_1\varepsilon_s \\ u_2\varepsilon_s \end{pmatrix}, \tag{S13}
$$

where C is a normalization constant, and $\varepsilon_s = (1, si)/\sqrt{2}$ with $s = \pm$ refers to $p_x \pm ip_y$ orbitals. The relative sign between u_1 and u_2 determines whether the state is in-phase or out-of-phase. For $u_1u_2 > 0$ ($u_1u_2 < 0$) the eigenvector represents in-phase (out-of-phase) states. For the higher frequency mode, u_2 is always positive. Thus, the in-phase (out-of-phase) states have higher frequencies when $d_{AB} > 0$ ($d_{AB} < 0$). At Γ point, d_{AB} is always negative as required by the acoustic sum rule. At other high symmetry points, the positive value of d_{AB} indicates a band inversion between in-phase and out-of-phase modes. In Tab. S2 we showed the coupling parameters that induce band inversions at K or A, which gives type-I and type-II phononic TIs or TSMs discussed in the main text. We used the lattice parameters$c_1 = c_2 = \sqrt{3}a$.

IV. HYBRID WANNIER CENTERS

If a band does not hybridize with all other bands throughout the Brillouin zone (BZ), it is called isolated. For an isolated phonon band belonging to the nth branch, i.e. $D_{\mathbf{k}}|\mathbf{u}_{n\mathbf{k}}\rangle = \omega_{n\mathbf{k}}^2|\mathbf{u}_{n\mathbf{k}}\rangle$, its Wannier center along the z-axis can be calculated through the Berry phase as

$$
z_n(k_x, k_y) = \frac{c}{2\pi}\phi_n(k_x, k_y), \tag{S14}
$$

where $\phi_n(k_x, k_y) = \int_{-\pi/c}^{\pi/c} A_n^z\, dk_z$ is the Berry phase, $A_n^z = \langle\mathbf{u}_{n\mathbf{k}}| - i\partial_{k_z}|\mathbf{u}_{n\mathbf{k}}\rangle$ is the Berry connection, and c is the lattice constant along the z-axis. If a phonon band is not isolated, its Wannier function will hybridize with some other bands. In this case we should find a set of hybridized bands that are isolated from the others. Then the Berry phase $\phi_n(k_x, k_y)$ in Eq. (S14) should be

TABLE S1: Dynamical matrix of Eq. (S11) at high symmetry points, where $\alpha = A, B$.

	D_α	D_{AB}
Γ	$[k^T_{1AB} + 6k^T_{2AB} + 3(\tilde{k}^L_{2AB} - \tilde{k}^T_{2AB})$ $+k^T_{1BA} + 6k^T_{2BA} + 3(\tilde{k}^L_{2BA} - \tilde{k}^T_{2BA})]\mathbb{I}_2$	$[-k^T_{1AB} - 6k^T_{2AB} - 3(\tilde{k}^L_{2AB} - \tilde{k}^T_{2AB})$ $-k^T_{1BA} - 6k^T_{2BA} - 3(\tilde{k}^L_{2BA} - \tilde{k}^T_{2BA})]\mathbb{I}_2$
K	$\frac{9}{2}(k^L_{1\alpha} + k^T_{1\alpha})\mathbb{I}_2 + D_\alpha(\Gamma)$	$[-k^T_{1AB} + 3k^T_{2AB} + \frac{3}{2}(\tilde{k}^L_{2AB} - \tilde{k}^T_{2AB})$ $-k^T_{1BA} + 3k^T_{2BA} + \frac{3}{2}(\tilde{k}^L_{2BA} - \tilde{k}^T_{2BA})]\mathbb{I}_2$
M	$4(k^L_{1\alpha} + k^T_{1\alpha})\mathbb{I}_2 - 2(k^L_{1\alpha} - k^T_{1\alpha})\sigma_z + D_\alpha(\Gamma)$	$(-k^T_{1AB} + 2k^T_{2AB})\sigma_z + (\tilde{k}^L_{2AB} - \tilde{k}^T_{2AB})(\mathbb{I}_2 - 2\sigma_z)$ $(-k^T_{1BA} + 2k^T_{2BA})\mathbb{I}_2 + (\tilde{k}^L_{2BA} - \tilde{k}^T_{2BA})(\mathbb{I}_2 - 2\sigma_z)$
A	$[k^T_{1AB} + 6k^T_{2AB} + 3(\tilde{k}^L_{2AB} - \tilde{k}^T_{2AB})$ $k^T_{1BA} + 6k^T_{2BA} + 3(\tilde{k}^L_{2BA} - \tilde{k}^T_{2BA})]\mathbb{I}_2$	$k^T_{1AB} + 6k^T_{2AB} + 3(\tilde{k}^L_{2AB} - \tilde{k}^T_{2AB})$ $-k^T_{1BA} - 6k^T_{2BA} - 3(\tilde{k}^L_{2BA} - \tilde{k}^T_{2BA})]\mathbb{I}_2$
H	$\frac{9}{2}(k^L_{1\alpha} + k^T_{1\alpha})\mathbb{I}_2 + D_\alpha(\Gamma)$	$[k^T_{1AB} - 3k^T_{2AB} - \frac{3}{2}(\tilde{k}^L_{2AB} - \tilde{k}^T_{2AB})$ $-k^T_{1BA} + 3k^T_{2BA} + \frac{3}{2}(\tilde{k}^L_{2BA} - \tilde{k}^T_{2BA})]\mathbb{I}_2$
R	$4(k^L_{1\alpha} + k^T_{1\alpha})\mathbb{I}_2 - 2(k^L_{1\alpha} - k^T_{1\alpha})\sigma_z + D_\alpha(\Gamma)$	$(k^T_{1AB} - 2k^T_{2AB})\mathbb{I}_2 - (\tilde{k}^L_{2AB} - \tilde{k}^T_{2AB})(\mathbb{I}_2 - 2\sigma_z)$ $(-k^T_{1BA} + 2k^T_{2BA})\mathbb{I}_2 + (\tilde{k}^L_{2BA} - \tilde{k}^T_{2BA})(\mathbb{I}_2 - 2\sigma_z)$

TABLE S2: Values of coupling parameters for type-I phononic TI/TSM and type-II phononic TI.

	k^L_{1A}	k^T_{1A}	k^L_{1B}	k^T_{1B}	k^L_{1AB}	k^T_{1AB}	k^L_{1BA}	k^T_{1BA}	k^L_{2AB}	k^T_{2AB}	k^L_{2BA}	k^T_{2BA}
Type-I TI	1	0.7	0.6	1.2	0	0	0	6.5	0	2.5	0	2.5
Type-I TSM	1	1	1	1	0	0	0	6.5	0	2.5	0	2.5
Type-II TI	0	2	2	0	0	8	0	8	0	4	1	0

TABLE S3: Values of matrix element d_{AB} at high symmetry points for type-I phononic TI/TSM and type-II phononic TI.

	Γ	K	A	H
Type-I TI	−32.75	+6.625	−6.5	−6.5
Type-I TSM	−32.75	+6.625	−6.5	−6.5
Type-II TI	−37.75	−5.125	+20.25	−10.125

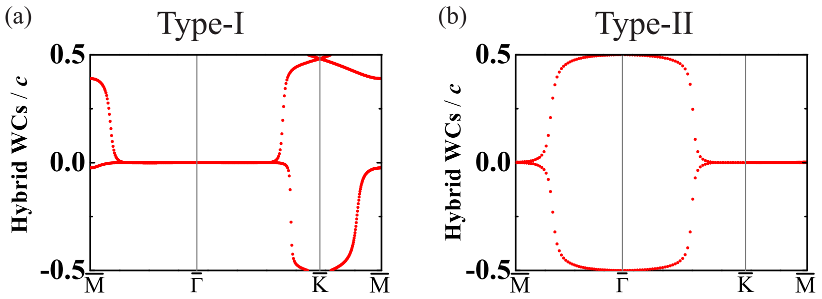

FIG. S2: Hybrid Wannier centers for type-I (a) and type-II (b) phononic TIs.

replaced by the eigenvalue of Berry phase matrix $\Phi(k_x, k_y)$, whose matrix elements are given by $\Phi_{nm}(k_x, k_y) = \int_{-\pi/c}^{\pi/c} A^z_{nm}\, dk_z$, where $A^z_{nm} = \langle \mathbf{u}_{n\mathbf{k}}| - i\partial_{k_z}|\mathbf{u}_{m\mathbf{k}}\rangle$ is the non-Abelian Berry connection between the hybridized bands n and m.

For type-I and type-II phononic TIs, the bands below the topological band gaps form an isolated set. Their hybrid Wannier centers are calculated and shown in Fig. S2.

V. PSEUDOSPIN TEXTURES

Solutions to effective Hamiltonians.—The type-I effective Hamiltonian in Eq. (S6) can be divided into three parts: the diagonal part $H_0 = \text{diag}\{M_\mathbf{k}, -M_\mathbf{k}, M_\mathbf{k}, -M_\mathbf{k}\}$, the intrinsic pseudo-SOC part H_I, and the Rashba-Dresselhause pseudo-SOC part H_{RD}. The

solution to H_0 is the pseudospin polarized states. In the basis of $\{|\mathrm{I}_\uparrow\rangle, |\mathrm{O}_\uparrow\rangle, |\mathrm{I}_\downarrow\rangle, |\mathrm{O}_\downarrow\rangle\}$ its solutions can be expressed as:

$$\begin{aligned}
\omega_1^{(0)} &= M_{\mathbf{k}} : |\phi_1^{(0)}\rangle = |\mathrm{I}_\uparrow\rangle = (1,0,0,0)^T \\
\omega_2^{(0)} &= -M_{\mathbf{k}} : |\phi_2^{(0)}\rangle = |\mathrm{O}_\uparrow\rangle = (0,1,0,0)^T \\
\omega_3^{(0)} &= M_{\mathbf{k}} : |\phi_3^{(0)}\rangle = |\mathrm{I}_\downarrow\rangle = (0,0,1,0)^T \\
\omega_4^{(0)} &= -M_{\mathbf{k}} : |\phi_4^{(0)}\rangle = |\mathrm{O}_\downarrow\rangle = (0,0,0,1)^T
\end{aligned} \tag{S15}$$

We treat H_I and H_{RD} as perturbations. The correction of H_{RD} can be addressed through degenerate perturbation theory:

$$\begin{aligned}
\delta\omega_1 &= C_1 k : |\phi_1\rangle = -\frac{ik_+}{\sqrt{2}k}|\phi_1^{(0)}\rangle + \frac{1}{\sqrt{2}}|\phi_3^{(0)}\rangle, \\
\delta\omega_2 &= C_2 k : |\phi_2\rangle = \frac{ik_-}{\sqrt{2}k}|\phi_2^{(0)}\rangle + \frac{1}{\sqrt{2}}|\phi_4^{(0)}\rangle, \\
\delta\omega_3 &= -C_1 k : |\phi_3\rangle = -\frac{ik_+}{\sqrt{2}k}|\phi_1^{(0)}\rangle - \frac{1}{\sqrt{2}}|\phi_3^{(0)}\rangle, \\
\delta\omega_4 &= -C_2 k : |\phi_4\rangle = \frac{ik_-}{\sqrt{2}k}|\phi_2^{(0)}\rangle - \frac{1}{\sqrt{2}}|\phi_4^{(0)}\rangle.
\end{aligned} \tag{S16}$$

The correction of H_I can be addressed through non-degenerate perturbation theory:

$$\begin{aligned}
\delta\omega_1 &= \frac{\Delta_1^2}{2M_{\mathbf{k}}}(k_x^2+k_y^2) + \frac{\Delta_2^2 k_z^2}{2M_{\mathbf{k}}} : |\psi_1\rangle = |\phi_1\rangle + \frac{\Delta_1 k_-}{2M_{\mathbf{k}}}|\phi_2\rangle + \frac{\Delta_2 k_z}{2M_{\mathbf{k}}}|\phi_4\rangle, \\
\delta\omega_2 &= -\frac{\Delta_1^2}{2M_{\mathbf{k}}}(k_x^2+k_y^2) - \frac{\Delta_2^2 k_z^2}{2M_{\mathbf{k}}} : |\psi_2\rangle = |\phi_2\rangle - \frac{\Delta_1 k_+}{2M_{\mathbf{k}}}|\phi_1\rangle - \frac{\Delta_2 k_z}{2M_{\mathbf{k}}}|\phi_3\rangle, \\
\delta\omega_3 &= \frac{\Delta_1^2}{2M_{\mathbf{k}}}(k_x^2+k_y^2) + \frac{\Delta_2^2 k_z^2}{2M_{\mathbf{k}}} : |\psi_3\rangle = |\phi_3\rangle + \frac{\Delta_2 k_z}{2M_{\mathbf{k}}}|\phi_2\rangle - \frac{\Delta_1 k_+}{2M_{\mathbf{k}}}|\phi_4\rangle, \\
\delta\omega_4 &= -\frac{\Delta_1^2}{2M_{\mathbf{k}}}(k_x^2+k_y^2) - \frac{\Delta_2^2 k_z^2}{2M_{\mathbf{k}}} : |\psi_4\rangle = |\phi_4\rangle - \frac{\Delta_2 k_z}{2M_{\mathbf{k}}}|\phi_1\rangle - \frac{\Delta_1 k_-}{2M_{\mathbf{k}}}|\phi_3\rangle.
\end{aligned} \tag{S17}$$

For type-II effective Hamiltonian, the solutions are given by replacing $k_\pm$ by $k_\mp^2$ in Eq. (S17).

Definition of pseudospin textures.—We have introduced a pseudospin index σ_z to descriminate the phonon modes at high symmetry points at K, H and Γ, A. Away from these high symmetry points, σ_z is not conserved. We introduce other two components of pseudospin vectors in analogy to electron spins:

$$\begin{aligned}
\sigma_x &= \sum_{a=\mathrm{I,O}} |a_\uparrow\rangle\langle a_\downarrow| + |a_\downarrow\rangle\langle a_\uparrow|, \\
\sigma_y &= \sum_{a=\mathrm{I,O}} -i|a_\uparrow\rangle\langle a_\downarrow| + i|a,\downarrow\rangle\langle a_\uparrow|,
\end{aligned} \tag{S18}$$

where the summation is over in-phase and out-of-phase modes. In the basis of $\{|\mathrm{I}_\uparrow\rangle, |\mathrm{O}_\uparrow\rangle, |\mathrm{I}_\downarrow\rangle, |\mathrm{O}_\downarrow\rangle\}$, the x, y components of pseudospin vectors can be expressed as

$$\sigma_x = \begin{pmatrix} 0 & 0 & 1 & 0 \\ 0 & 0 & 0 & 1 \\ 1 & 0 & 0 & 0 \\ 0 & 1 & 0 & 0 \end{pmatrix}, \qquad \sigma_y = \begin{pmatrix} 0 & 0 & -i & 0 \\ 0 & 0 & 0 & -i \\ i & 0 & 0 & 0 \\ 0 & i & 0 & 0 \end{pmatrix}. \tag{S19}$$

For a given $\mathbf{k}$ point, the pseudospin polarization can be calculated as:

$$\begin{aligned}
\langle\sigma_x^i\rangle &= \langle\psi_i|\sigma_x|\psi_i\rangle, \\
\langle\sigma_y^i\rangle &= \langle\psi_i|\sigma_y|\psi_i\rangle, \\
\langle\sigma_z^i\rangle &= \langle\psi_i|\sigma_z|\psi_i\rangle,
\end{aligned} \tag{S20}$$

where $i = 1, 2, 3, 4$ refers to different bands.

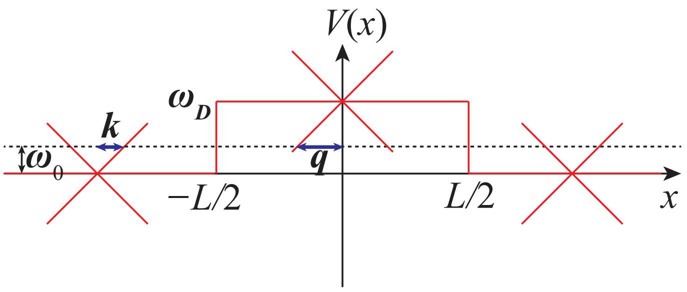

FIG. S3: Schematic of a tunneling barrier for TSSs.

VI. PHONON TRANSPORT IN TUNNELING JUNCTIONS

Transmission of type-I topological surface states.—For type-I topological surface states (TSSs), the effective Hamiltonian can be expressed as

$$H_{\text{surf}} = v_D \begin{pmatrix} 0 & -i\partial_x - ik_y \\ -i\partial_x + ik_y & 0 \end{pmatrix} + V(x), \tag{S21}$$

where

$$V(x) = \begin{cases} \omega_D & |x| > L/2 \\ 0 & |x| \leq L/2 \end{cases}. \tag{S22}$$

ω_D and L is the height and width of the tunneling junction, respectively [Fig. S3]. Here we have replaced k_x by $-i\partial_x$ and consider only the $\bar{K}$ valley state ($\tau_z = 1$) by neglecting intervalley scattering. The solution to the incident state is $H_{\text{surf}}\psi^i = \omega_0\psi^i$, where $\omega_0 = v_D k$ is the incident wave frequency (referenced to the Dirac point).

$$\psi^i = \frac{1}{\sqrt{2}k} \begin{pmatrix} k_x - ik_y \\ k \end{pmatrix} e^{i(k_x x + k_y y)} = \frac{1}{\sqrt{2}} \begin{pmatrix} e^{-i\theta} \\ 1 \end{pmatrix} e^{i(k_x x + k_y y)}, \tag{S23}$$

where $k = \sqrt{k_x^2 + k_y^2}$ is the incident wave vector, and $\theta = \arctan(k_y/k_x)$ is the incident angle. For the reflected state k_x should be changed to $-k_x$ i.e.

$$\psi^r = \frac{r}{\sqrt{2}k} \begin{pmatrix} -k_x - ik_y \\ k \end{pmatrix} e^{i(-k_x x + k_y y)} = \frac{r}{\sqrt{2}} \begin{pmatrix} -e^{i\theta} \\ 1 \end{pmatrix} e^{i(-k_x x + k_y y)}, \tag{S24}$$

where r refers to the reflection coefficient. The state inside the barrier is

$$\tilde{\psi} = \frac{a}{\sqrt{2}} \begin{pmatrix} e^{-i\varphi} \\ -1 \end{pmatrix} e^{i(q_x x + q_y y)} + \frac{b}{\sqrt{2}} \begin{pmatrix} -e^{i\varphi} \\ -1 \end{pmatrix} e^{i(-q_x x + q_y y)}, \tag{S25}$$

which contains both left-moving and right-moving states. In the central barrier region, the wave vector $\mathbf{q}$ is given by $v_D\sqrt{q_x^2 + q_y^2} = \omega_D - \omega_0$ and the angle $\varphi = \arctan(q_y/q_x)$. The transmitted state is

$$\psi^t = \frac{t}{\sqrt{2}} \begin{pmatrix} e^{-i\theta} \\ -1 \end{pmatrix} e^{i(k_x x + k_y y)}. \tag{S26}$$

By matching the wave functions at the boundary $x = -L/2$ and $x = L/2$,

$$\begin{aligned} \psi^i|_{x=-L/2} + \psi^r|_{x=-L/2} &= \tilde{\psi}|_{x=-L/2}, \\ \tilde{\psi}|_{x=L/2} &= \psi^t|_{x=L/2}. \end{aligned} \tag{S27}$$

We can solve the equations for the variables a, b, r, and t, for given ω_D, L, and θ. Then we can calculate the phonon transmission

$$T = |t|^2. \tag{S28}$$

Parameters in the calculations of Figs. 3(b)-(c) are $v_D = 1$, $\omega_0 = 0.1$, $\omega_D = 0.3$.

Transmission of type-II topological surface states.—For type-II TSSs, the surface effective Hamiltonian is

$$H'_{\rm surf} = D\begin{pmatrix} 0 & (-i\partial_x + ik_y)^2 \\ (-i\partial_x - ik_y)^2 & 0 \end{pmatrix} + V(x). \tag{S29}$$

The incident and reflected states can be expressed as

$$\psi^i = \frac{1}{\sqrt{2}}\begin{pmatrix} e^{2i\theta} \\ -1 \end{pmatrix} e^{i(k_x x + k_y y)}, \psi^r = \frac{r}{\sqrt{2}}\begin{pmatrix} e^{-2i\theta} \\ -1 \end{pmatrix} e^{i(-k_x x + k_y y)}. \tag{S30}$$

In addition to the ordinary incident and reflected states, the type-II effective Hamiltonian has an edge state solution for $x \leq -L/2$:

$$\psi_1^{\rm edge} = a\begin{pmatrix} -(\sqrt{1+\sin^2\theta} - \sin\theta)^2 \\ 1 \end{pmatrix} e^{\alpha x + ik_y y}, \tag{S31}$$

where $\alpha = \sqrt{k_x^2 + 2k_y^2}$. This solution does not exist for the type-I surface Hamiltonian $H_{\rm surf}$. The state inside the barrier is

$$\tilde{\psi} = b\begin{pmatrix} e^{2i\varphi} \\ 1 \end{pmatrix} e^{i(q_x x + q_y y)} + c\begin{pmatrix} e^{-2i\varphi} \\ 1 \end{pmatrix} e^{i(-q_x x + q_y y)} + d\begin{pmatrix} (\sqrt{1+\sin^2\varphi} - \sin\varphi)^2 \\ 1 \end{pmatrix} e^{\beta x + iq_y y} + f\begin{pmatrix} 1 \\ (\sqrt{1+\sin^2\varphi} - \sin\varphi)^2 \end{pmatrix} e^{-\beta x + iq_y y}, \tag{S32}$$

where $\beta = \sqrt{q_x^2 + 2q_y^2}$ and $\varphi = \arctan(q_y/q_x)$. The transmitted and edge states for $x \geq L/2$ are

$$\psi^t = \frac{t}{\sqrt{2}}\begin{pmatrix} e^{2i\theta} \\ 1 \end{pmatrix} e^{i(k_x x + k_y y)}, \psi_2^{\rm edge} = g\begin{pmatrix} 1 \\ -(\sqrt{1+\sin^2\theta} - \sin\theta)^2 \end{pmatrix} e^{-\alpha x + iq_y y}. \tag{S33}$$

The transmission coefficient t and the transmission $T = |t|^2$ can be solved by matching the wave functions and their derivatives at the two boundaries:

$$\begin{aligned} (\psi^i + \psi^r + \psi_1^{\rm edge})|_{x=-L/2} &= \tilde{\psi}|_{x=-L/2}, \\ (\partial_x\psi^i + \partial_x\psi^r + \partial_x\psi_1^{\rm edge})|_{x=-L/2} &= \partial_x\tilde{\psi}|_{x=-L/2}, \\ \tilde{\psi}|_{x=L/2} &= (\psi^t + \psi_2^{\rm edge})|_{x=L/2}, \\ \partial_x\tilde{\psi}|_{x=L/2} &= (\partial_x\psi^t + \partial_x\psi_2^{\rm edge})|_{x=L/2}. \end{aligned} \tag{S34}$$

Parameters in the calculations of Figs. 3(b)-(c) are $D = 1$, $\omega_0 = 0.1$, $\omega_D = 0.3$.

Transmission of normal surface states.—Normal surface states without pseudospin-momentum locking can be described by a one-component wave function. For simplicity, a linear dispersion ($\omega_{\mathbf{k}} = \pm\sqrt{k_x^2 + k_y^2}$) the same as the type-I TSSs is selected for the normal surface states. The incident and reflected states are

$$\psi^i = e^{i(k_x x + k_y y)}, \psi^r = re^{i(-k_x x + k_y y)}. \tag{S35}$$

The states inside the barrier are

$$\tilde{\psi} = ae^{i(q_x x + q_y y)} + be^{i(-q_x x + q_y y)}. \tag{S36}$$

The transmitted state is

$$\psi^t = te^{i(k_x x + k_y y)}. \tag{S37}$$

The transmission coefficient t can be solved by matching the wave functions and their derivatives at the two boundaries $x = \pm L/2$. Parameters in the calculations of Figs. 3(b)-(c) are $\omega_0 = 0.1$, $\omega_D = 0.3$.

VII. INFLUENCE OF OUT-OF-PLANE VIBRATIONS

We include out-of-plane vibrations in phonon calculations to see their influence on topological properties of in-plane vibrations. Phonon dispersion curves of a type-I phononic TI including out-of-plane vibrations are shown in Fig. S4(a). The projection onto the in-plane in-phase and out-of-phase modes are also calculated. Warmer colors indicate higher values of $|\langle \mathbf{u}_{\rm out}|\mathbf{u}_{\mathbf{k}}\rangle|^2 - |\langle \mathbf{u}_{\rm in}|\mathbf{u}_{\mathbf{k}}\rangle|^2$, where $|\mathbf{u}_{\rm in}\rangle = \frac{1}{2}(1, \pm i, 0, 1, \pm i, 0)^T$ and $|\mathbf{u}_{\rm out}\rangle = \frac{1}{2}(1, \pm i, 0, -1, \mp i, 0)^T$ refer to the in-plane I and O states, respectively. The two dispersion curves colored in green correspond to the out-of-plane modes which do not appear within the topological band gap thus do not couple with the TSSs. Therefore the band topology of type-I phononic TI is unaffected by the out-of-plane vibrations. This is verified in our surface-state calculations including out-of-plane vibrations shown in Fig. S4(b).

For type-II phononic TI, phonon dispersion curves including out-of-plane vibrations are shown in Fig. S4(c). Out-of-plane modes do not appear within the topological band gap neither. Figure S4(d) shows the type-II TSSs when out-of-plane vibrations are included.

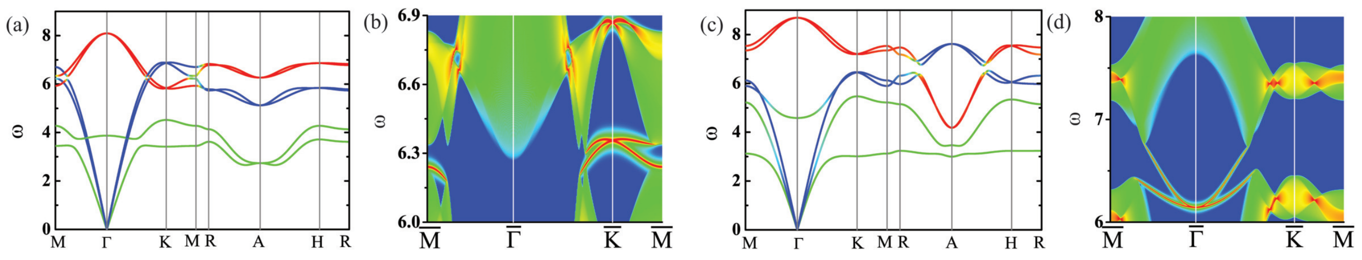

FIG. S4: (a) Dispersion curves of a type-I phononic TI including out-of-plane vibrations. Blue (red) color is used to denote the contribution of in-plane I (O) vibration modes. (b) LDOS of the (001) surface including both in-plane and out-of-plane vibrations, where higher (lower) LDOS are colored red (blue). (c) Dispersion curves of a type-II phononic TI including out-of-plane vibrations. (d) LDOS of the (001) surface including both in-plane and out-of-plane vibrations.

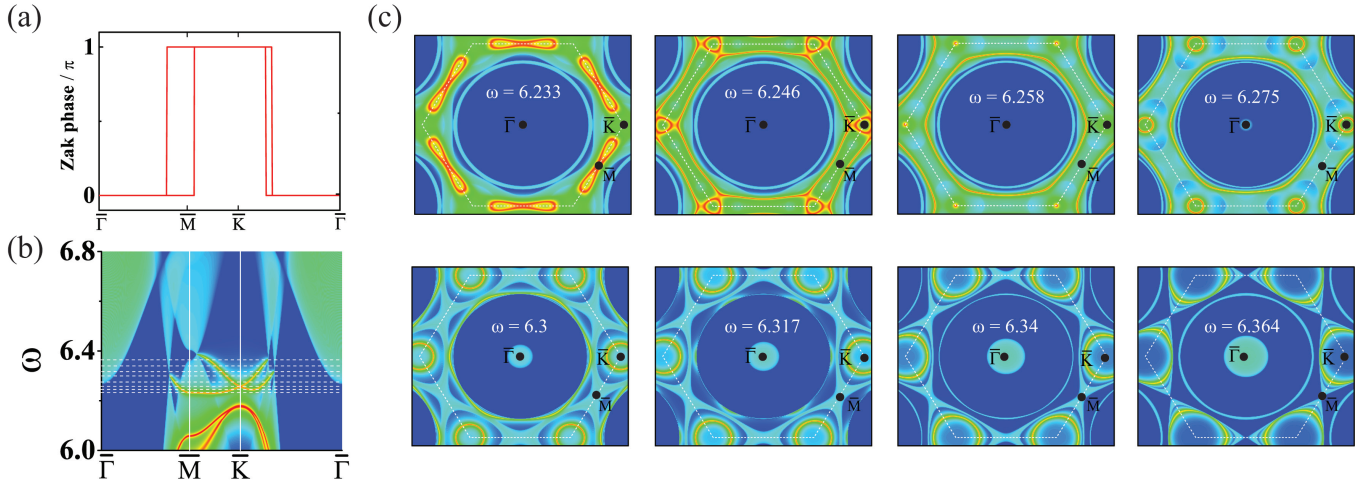

FIG. S5: (a) Zak phases along the (001) direction of the lowest two bands for a type-I phononic TSM. (b) LDOS of the (001) surface, where higher (lower) LDOS are colored red (blue). (c) LDOS of the (001) surface for varying equifrequency planes indicated by the white dashed lines in (b), showing the evolution of drumhead surface states with varying phonon frequencies.

VIII. PHONONIC TOPOLOGICAL SEMIMETAL

The calculated Zak phases along the (001) direction of the lowest two bands are shown in Fig. S5(a). The drumhead surface states were shown in Fig. S5(b). Figure S5(c) shows the evolution of the drumhead surface states with varying phonon frequencies.
